# Giant urethral calculus in anterior urethral diverticulum: a case report

**DOI:** 10.1186/s12894-019-0498-9

**Published:** 2019-07-29

**Authors:** Maokun Sun, Wanting Xu, Shuai Guo, Wenyi Ma, Haiyue Xu, Ruili Sun

**Affiliations:** 1 Department of Urology, Liaocheng People’s Hospital, Liaocheng, Shandong China; 2 Liaocheng Food and Drug Administration, Liaocheng, Shandong China; 3 Division of Molecular Pharmaceutics and Drug Delivery, The University of Texas at Austin, College of Pharmacy, Austin, TX USA

**Keywords:** Urethral calculus, Lithotripsy, Diverticulum

## Abstract

**Background:**

In this case report, giant calculus in the urethral diverticulum was found through ureteroscopy investigation, the pneumatic lithotripsy combined with ultrasound lithotripsy (PLCUL) was successfully performed to break down this rare and giant urethral calculus in the diverticulum without open surgery.

**Case presentation:**

A 82-year-old male presented to the urology department, complaining of frequent urination and dysuria. One giant, dark brown stone (6.5 × 6 × 5.5 cm) was revealed in the diverticulum of the anterior urethra using combination of local ultrasound, pelvic Computer Tomography (CT) and Magnetic Resonance Imaging (MRI). The stone was then successfully broken down via the PLCUL, and the emptied anterior urethral diverticulum was left untreated. In the 18 months’ follow-up, no new calculus was found in urethral tract, anterior diverticula became gradually smaller, eventually disappeared.

**Conclusion:**

In the treatment of giant calculus in the urethral diverticulum, this case report provides an effective method of lithotripsy in the clinical trials.

## Background

Urethral calculus in male often cause dysuria with urinary tract irritation and acute urinary retention, which is considered as a urological emergency. Usually, small stones can be discharged naturally. Those small ones located at the anterior or more deep positions in urinary tract can be squeezed out followed by application of lubricants with low pressure injection. If the procedure fails, open surgical interventions may be adopted. For the huge anterior urethral diverticulum with urethral calculus, open surgery (i.e. diverticulum resection and urethrostomy) is conventional approach. Krystian [1] performed one open surgery to remove the urethral calculus and the following urethroplasty in a previous reported case of urethral giant stones complicated with multiple urethral stricture due to multiple pelvic fracture. Clinical results showed the postoperative recovery was very good. Nonetheless, for the open surgery procedure, patients always undergo major open wounds and slow recovery, associated with the risk of urinary tract infections, stenosis and urinary fistula complications. In this case, a rare giant urethral calculus was found in the urethral diverticulum of a male patient, PLCUL technique was performed to break down the giant urethral calculus in the diverticulum without open surgery, this case study provides a feasible and effective method in the treatment of giant calculus in the urethral diverticulum.

## Case presentation

**Fig. 1:**
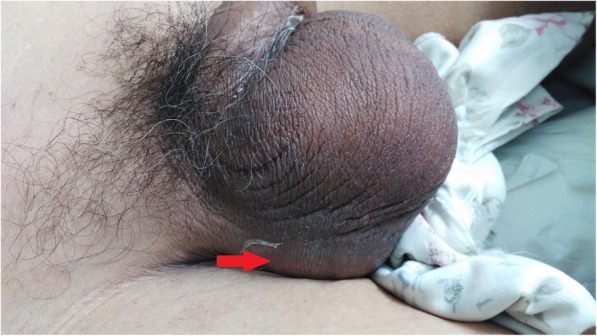
Physical examination showed swelling and tenderness located below the scrotal skin near the root of penis, the arrow indicates the position of calculus was underneath the skin

**Fig. 2:**
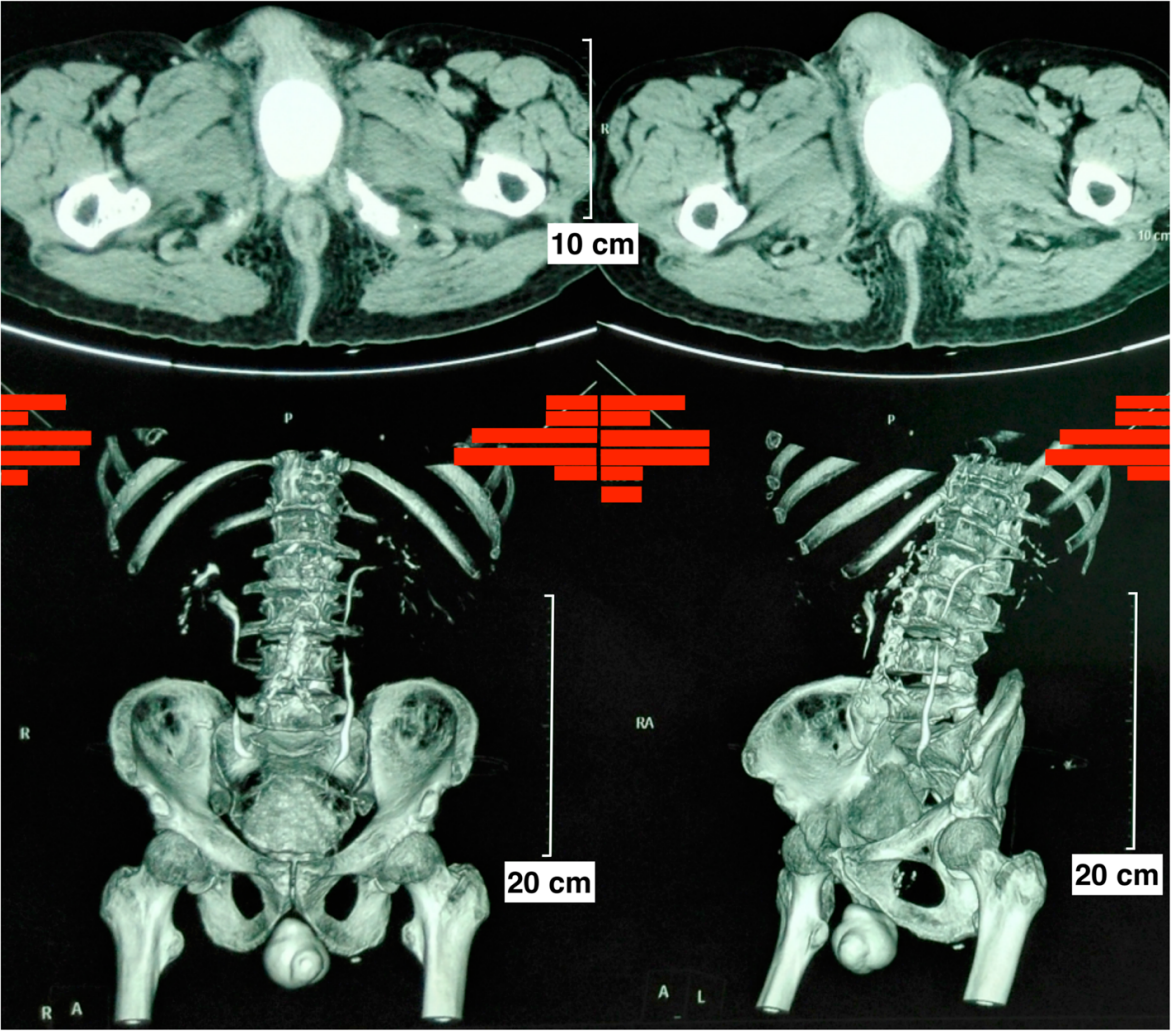
A giant urethral calculus with a size of 6.5 × 6 × 5.5 cm was observed below the pubic symphysis in the enhanced urinary CT

**Fig. 3:**
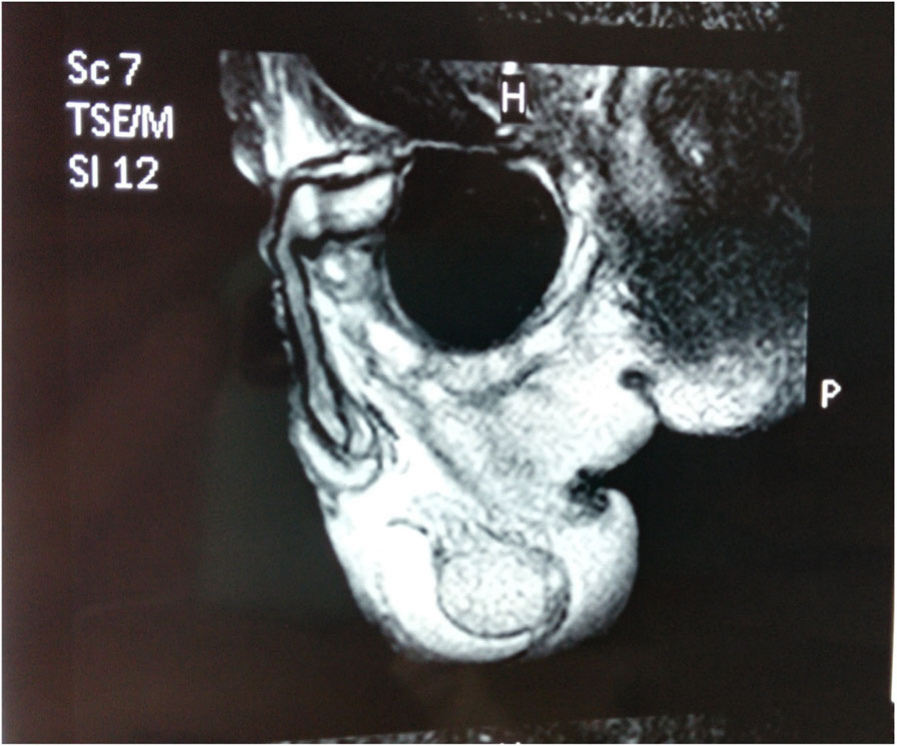
Irregular clumps was observed in the subcutaneous posterior scrotum in the prostate MR scan before lithotripsy

**Fig. 4:**
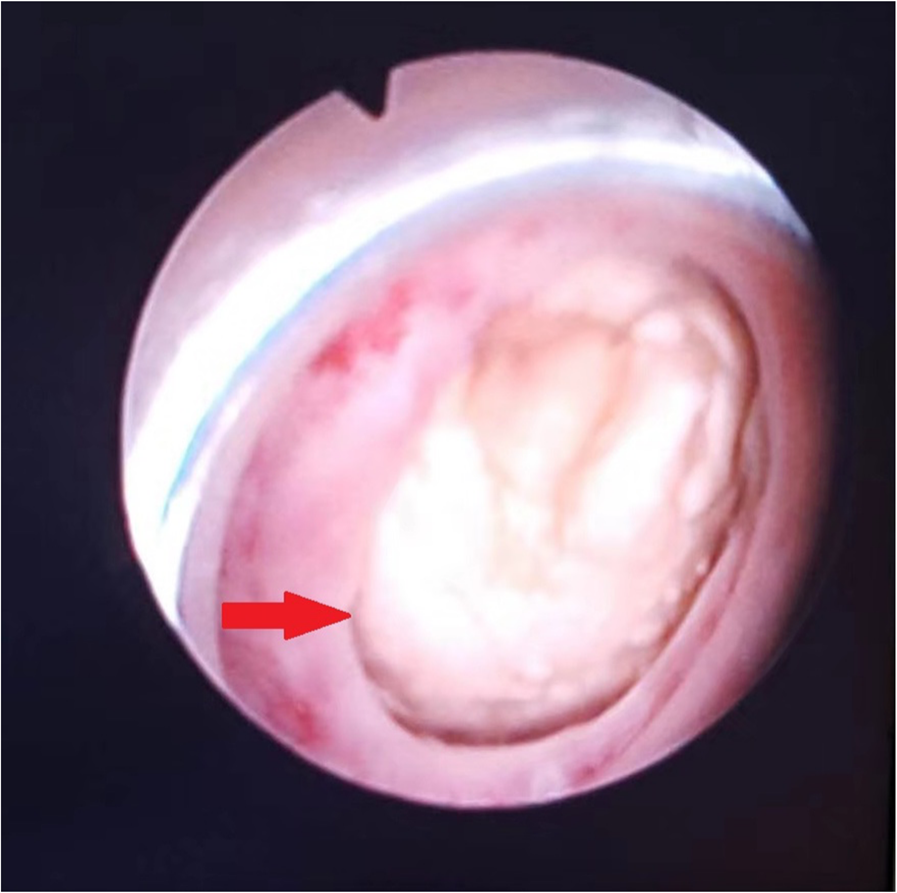
A giant, dark brown stone was revealed to located in the urethral diverticulum through ureteroscopy investigation, the arrow indicates the calculus in the urethral diverticulum

A 82-year-old male presented to the urological department, complaining of swelling and pain in perineal region, along with a frequent, painful, and difficult urination, when initially started 6 years ago. The patient was diagnosed with prostatitis and treated with quinolone antibiotics in several local hospitals. However, the symptoms did not ease and the patient showed up in our hospital where he was referred to our urology department. After multiple urinalysis, it was confirmed the patient had urinary tract infection. Blood parameters, biochemical, coagulation mechanisms, virus screening and other indicators were in normal level. In the physical examination, swelling and tenderness were observed in bilateral scrotum and perineum. A hard mass with a size of 7 × 7 × 6 cm was detected below the scrotal skin near the root of penis. The surrounding tissue boundaries of the mass was not clear. The patient experienced pain during the physical examination of the mass (Fig. 1). In the enhanced urinary CT scan, a compact shadow of round shape with a size of 6.5 × 6 × 5.5 cm was observed below the pubic symphysis. Without a significant enhancement, the shadow has a clear border and uniform density in the scan. Enlargement of the scrotal volume was also observed. A disorder structure of internal scrotum with multiple low-density liquid regions was detected (Fig. 2). In the MRI scan of prostate gland, irregular clumps of T1 and T2 signals was observed in the subcutaneous posterior scrotum. SPAIR signals was high and without a clear border. There were no obvious high signals in DWI. The prostate gland showed a normal morphology, without significant abnormal signals in the scan (Fig. 3). On the urethroscopy investigation, a giant, dark brown stone was revealed to be ventrally attached to the urethral wall, which is located at the rear of the urethral bulb (Fig. 4).

**Fig. 5:**
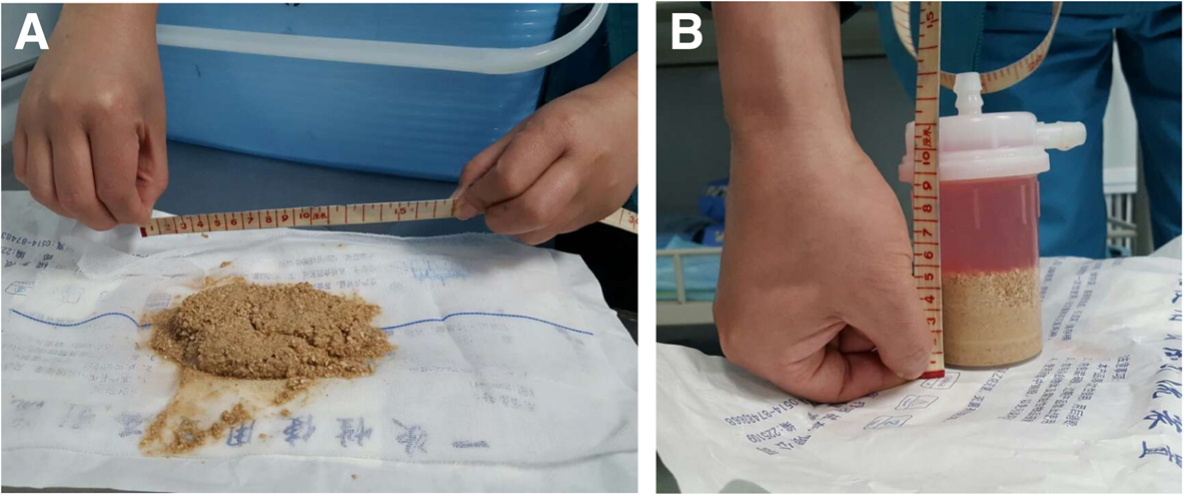
Urethral calculus was removed after the lithotripsy, the metric rulers within unit of centimeter (e.g. number 8 means 8 cm in length) indicate **a**) the estimate size of stone after break-down, and **b**) the estimated volume of shredded calculus was calculated as 100 mL

**Fig. 6:**
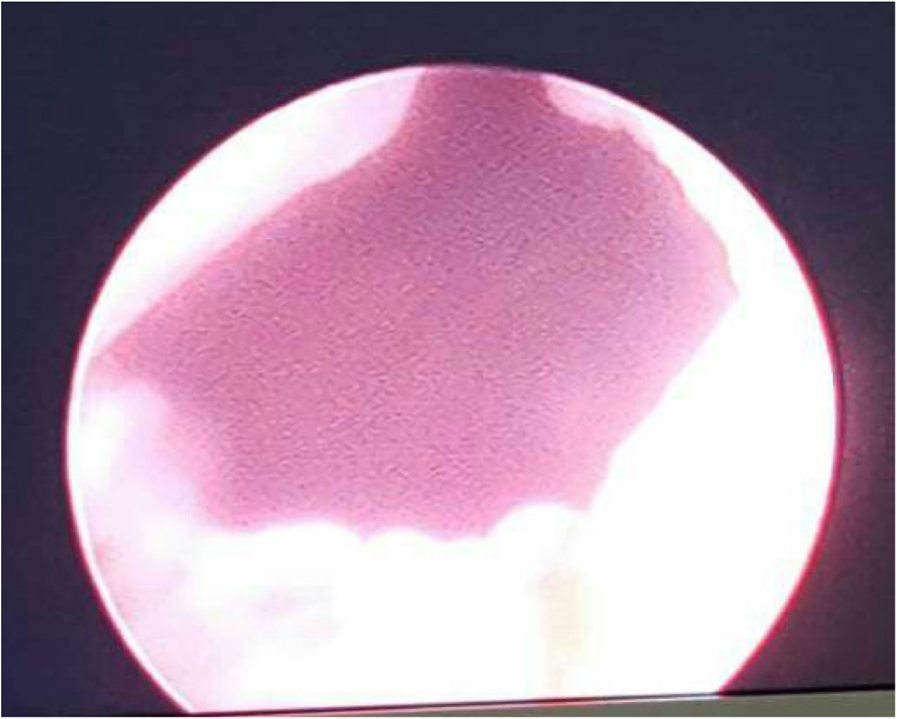
No residue of urethral calculus left in the urethral diverticulum after calculus removal through ureteroscopy investigation

The patient was treated with pneumatic lithotripsy combined with ultrasound lithotripsy (PLCUL) via LithoClast® Master (EMS, Nyon, Switzerland), the procedure lasted a total of 60 min and the amount of bleeding was about 15 ml. The stone was successfully broken down and removed (Figs. 5 and 6). Urethral diverticulum was left untreated. At the end of procedure, a catheter was left to support the urethra for 1 week. After the discharge, patient followed the doctors’ advice to squeeze urethral diverticulum to extrude residual urine to prevent the formation of new stones at the end of each urination. The patient was examined, for the presence of new stones formation or diverticulum changes, via ultrasound and urethraloscopy every 6 months in the outpatient clinic. Results showed that there was no formation of new stones in the urethral diverticulum and diverticula returned to the normal size (result not shown). The patient had normal functional urination until this paper is reported.

## Discussion and conclusions

Urethral calculus is always found on the site of prostatic urethra, bulbar and fossa navicularis. Koga [2], paulk [3] and Sharfi [4] reported most urethral stones occurred in the posterior urethra, whereas Amin [5] and Englisch [6] reported high occurrence in the anterior urethra. The majority of urethral calculus is actually kidney and bladder stones which accumulated or embedded in the urethra. However, there are a few occurrences of urethral calculus reported due to the urethral stricture, foreign body inside or primary urethral calculus in the urethra diverticulum. Urethral diverticulum is a protrusion of the urethra through the periurethral fascia [7]. In this case, the urethral diverticulum was considered to be caused by stones in the urinary tract, long-term stay of the urethral calculus led to local necrosis fibrosis, further to the formation of diverticula, along with repeated inflammations.

X-ray, urethrography, CT, MRI and urethral physical examination are usually used to confirm the presence of urethral calculus. In this case, urethral calculus and urethral diverticulum were basically diagnosed by physical examination (Fig. 1), CT (Fig. 2) and MRI (Fig. 3). In a further diagnosis, stones were clearly seen using cystoscopy (Fig. 4). This direct observation also helped to differentiate the tumor from the calculus in the urethra.

In the treatment of giant calculus in the urethral diverticulum, open surgery is commonly used in the clinical trials as described before. However, with advancement in technology and new equipment, endovascular treatment techniques are becoming more popular. Some authors reported the use of low energy extracorporeal shock wave lithotripsy (ESWL) and endoscopic ultrasonography in the treatment of urethral calculus. However, because the low energy ESWL equipped with the shock waves and X-ray radiation can cause damage to the surrounding tissue, it is not preferred in clinical trials. The heat generated by high-frequency vibration in endoscopic ultrasonography can also damage the surrounding tissue, the procedure also needs a lot of circulating water to cool sonophoretic probe, which is susceptible to damage during clinical use [8]. Various types of lasers were also used in clinical trials, but they were less efficient in treating large stones in urinary tract [9].

Pneumatic lithotripsy overcome the above shortcomings and suitable for almost all urinary tract stones. Pneumatic lithotripsy is the new lithotripsy technology invented in the 1990s [10]. It has a reliable lithotripsy function, minor tissue injury, no thermal effects, low cost of treatment and other advantages. The fourth generation of Electronic Systems Management (ESM) pneumatic system is equipped with ultrasound lithotripsy system to break down the stones, and the active negative pressure to clean the debris. PLCUL technique has high efficiency in the clinic trials. Olbert [11] reported that PLCUL significantly increased the clearance of renal stones. Also, Juan [12] used PLCUL in the treatment of the bladder stones. PLCUL has the advantages of less operating time, less tissue injury, less bleeding and higher lithotripsy efficiency than holmium laser treatment. Also, in our case report, PLCUL was successfully performed to treat the giant urethral calculus in the diverticulum (Figs. 5 and 6).

Urethral lithotripsy has been successfully performed with any type of efficient energy source: ballistic [13], ultrasonic [14], electrohydraulic [15], laser [16, 17], etc. The success rate for the endoscopic approach of urethral lithiasis is almost 100%, regardless if they are fragmented in situ, in the prostatic cavity, or in the urinary bladder [15]. For the complications associated with endoscopic surgery, injuries may vary from simple abrasions to false passages. It is necessary to carefully perform the extraction of larger stones or fragments of stones, as to ensure the compliance of the lumen in order to avoid tearing or avulsion of the urethral wall [18]. Bleeding may also be a consequence of parietal injury during lithotripsy, although it is usually clinically insignificant. The bleeding management requires insertion of a larger caliber urethra-vesical catheter [18]. In addition, the endoscopic treatment may also generate a urethral stricture, However, the incidence of this complication is low [18].

For the treatment of emptied urethral diverticulum after lithotripsy, if the open surgery is performed to remove the stone, the diverticulum resection and urethral modeling are usually performed. For endovascular calculus removal without open surgery, several conservative treatments on the emptied urethral diverticulum has been reported, such as urethral massage and prophylactic use of antibiotics, tamponade of cellulose or injection of teflon, but the effect was not as expected [19]. In our case report, the urethral diverticulum was located in the bulbous urethra and was easily touched by hand. After the removal of calculus, urethral diverticulum was left without treatment, the patient followed the doctors’ advice to massage the urethral diverticulum site after each urination by squeezing out the residual urine in the diverticular cavity. Eventually there was no new formation of urethral stones, the diverticula returned to the normal size.

## Data Availability

The supporting data and materials can be found in the file cabinet in the department of urology of Liaocheng People’s Hospital. These confidential patient data within the patient’s information should not be shared to the public. The datasets used and/or analysed during the current study are available from the corresponding author on reasonable request.

## Abbreviations

CT: Computer Tomography
ESM: Electronic Systems Management
ESWL: Extracorporeal Shock Wave Lithotripsy
MRI: Magnetic Resonance Imaging
PLCUL: Pneumatic lithotripsy combined with ultrasound lithotripsy
